# Dysfunctional Breathing in Children and Adults With Asthma

**DOI:** 10.3389/fped.2018.00406

**Published:** 2018-12-20

**Authors:** Gary J. Connett, Mike Thomas

**Affiliations:** University Hospital Southampton NHS Foundation Trust, Southampton, United Kingdom

**Keywords:** asthma, dysfunctioal breathing, paradoxical vocal fold motion, hyperventilation, breathing pattern disorders, inducible laryngeal obstruction, exercise inducible laryngeal obstruction, breathing retraining exercises

## Abstract

Asthma occurs across the life course. Its optimal treatment includes the use of personalized management plans that recognize the importance of co-morbidities including so-called “dysfunctional breathing.” Such symptoms can arise as a result of induced laryngeal obstruction (ILO) or alterations in the mechanics of normal breathing called breathing pattern disorders. Whilst these two types of breathing abnormalities might be related, studies tend to focus on only one of them and do not consider their relationship. Evidence for these problems amongst childhood asthmatics is largely anecdotal. They seem rare in early childhood. Both types are more frequently recognized in the second decade of life and girls are affected more often. These observations tantalizingly parallel epidemiological studies characterizing the increasing prevalence and severity of asthma that also occurs amongst females after puberty. Exercise ILO is more common amongst adolescents and young adults. It should be properly delineated as it might be causally related to specific treatable factors. More severe ILO occurring at rest and breathing pattern disorders are more likely to be occurring within a psychological paradigm. Dysfunctional breathing is associated with asthma morbidity through a number of potential mechanisms. These include anxiety induced breathing pattern disorders and the enhanced perception of subsequent symptoms, cooling and drying of the airways from hyperventilation induced hyperresponsiveness and a direct effect of emotional stimuli on airways constriction via cholinergic pathways. Hyperventilation is the most common breathing pattern disorder amongst adults. Although not validated for use in asthma, the Nijmegen questionnaire has been used to characterize this problem. Studies show higher scores amongst women, those with poorly controlled asthma and those with psychiatric problems. Evidence that treatment with breathing retraining techniques is effective in a primary care population including all types of asthmatics suggests the problem might be more ubiquitous than just these high-risk groups. Future challenges include the need for studies characterizing all types of dysfunctional breathing in pediatric and adult patient cohorts and clearly defined, age appropriate, interventional studies. Clinicians caring for asthmatics in all age groups need to be aware of these co-morbidities and routinely ask about symptoms that suggest these problems.

## Introduction

For most of the time breathing occurs sub-consciously. A network of brain stem neurones at the level of the medulla and pons forms the respiratory center which initiates rhythmic contraction of the respiratory muscles whilst co-ordinating these movements with other activities, such as speaking and swallowing (1). As humans we are able to override this otherwise automatic process, for example by breath holding to dive underwater or to forcefully blow out candles. Breathing can also be impacted upon by emotions. For example, bouts of laughter can harmlessly interrupt normal respiration. However, in some people physiologically inappropriate hyperventilation, in response to feelings of agitation and anxiety, can lead to disabling symptoms and become a chronic problem (2). In others abnormal dysfunctional breathing patterns, such as trying to breath through an obstructed larynx, rapid, shallow breathing, irregular breathing and predominant upper-chest breathing might also lead to troublesome symptoms with or without associated hypocapnia. Identifying such problems as a co-morbidity complicating asthma can be difficult as there is considerable overlap in the types of symptoms that occur and a complex interrelationship (3).

In this article we will review how the asthma phenotype might be impacted upon by dysfunctional breathing problems throughout the life course.

## Definitions and Terminology

Dysfunctional breathing might usefully be regarded as an overarching term that is inclusive of problems that are either thoracic or laryngeal in nature (4). Whilst both types are reported as occurring amongst asthmatics, there are no studies addressing the extent to which they both occur within individual patients. This is despite prevalence studies suggesting that both are common and that therefore there should be considerable overlap. Unfortunately the use of the term dysfunctional can be stigmatizing for patients and especially in children where it might have inappropriate connotations about dysfunctional families and elements of abuse or neglect. To address such issues, breathing pattern disorders is a recently recommended term describing functional abnormalities in the mechanics of the diaphragm and intercostal muscles that result in inefficient breathing. Similarly, a joint task force addressing upper airway problems has recommended the term inducible laryngeal obstruction (ILO) rather than laryngeal dysfunction or paradoxical vocal cord motion to denote extrathoracic airway problems. ILO usefully encompasses obstruction occurring at supraglottic as well as glottic (vocal fold) levels as characterized at laryngoscopy (5).

The most commonly recognized breathing pattern disorder and the first to be described was hyperventilation in adults (6). More recently however, breathing pattern disorders have come to encompass a wider spectrum of breathing abnormalities. These include periodic deep sighing, thoracic dominant breathing, forced abdominal expiration and thoracic-abdominal asynchrony (7, 8). The extent to which these other problems occur amongst asthmatics is unknown.

## Pathophysiology

The pathophysiology of dysfunctional breathing disorders is incompletely understood. It has been suggested that the occurrence of a characteristic complex of symptoms that can include breathlessness, chest tightness, sighing, yawning, chest discomfort, general fatigue, anxiety and abdominal bloating, might usefully be regarded as a learnt conditioned response to some sort of emotional distress that has taken on physical manifestations (9). Those affected often do not recognize that their symptoms are arising in this way and that they might be “catastrophising” their health fearing serious underlying illness. The resulting interference with normal automatic control probably occurs at a sub-conscious level.

Somatisation symptoms typically resemble those of an illness the affected person is aware of, has, or has had in the past. In this context, it is well-recognized that those patients presenting with acute hyperventilation, commonly also have undetected co-existing asthma (10). Amongst known asthmatics however, the situation is far more complex because of the difficulties distinguishing true asthma from asthmatic symptoms induced by dysfunctional breathing. Hyperventilation for example, occurs in the vast majority of acute asthma attacks as demonstrated by low arterial carbon dioxide tensions (11). The mechanism whereby this happens might include stimulation of irritant and stretch receptors, anxiety mediated effects on cholinergic bronchosconstriction (12) and a hyperventilatory response to the perception of increased airways resistance (13). Hyperventilation *per se* might then further worsen true asthma through bronchosconstrictive cooling and airway dehydration.

ILO, the involuntary narrowing of the upper airway during inspiration, is similarly complex and multifactorial. Anxiety and psychological disorders are thought to be major contributory factors in the majority of adults who are affected and particularly when symptoms occur spontaneously at rest (14). Amongst younger asthmatics, exercise induced laryngeal obstruction (EILO) is more common and should usefully be regarded as a separate condition. It can co-exist with exercise-induced asthma and psychological co-morbidities are less predominant than in ILO. Causal factors include the aerodynamic effects of high inspiratory flow rates, neurally mediated laryngeal hyper-reactivity and environmental factors, such as inhaling cold air. Gastro-esophageal reflux has been implicated but this is very common in unaffected individuals of all ages and a causal relationship has not been proven (15). Upper airway symptoms of rhinitis and post-nasal drip might also be a contributory precipitant of upper airway closure but is also unproven (16).

## Epidemiology

The findings of the main studies to determine the presence of hyperventilation in asthmatics are summarized in Table 1. These studies have used the Nijmegen questionnaire (Table 2). This was originally developed as a screening tool for symptomatic hyperventilation syndrome and subsequently used as a continuous measure of the benefits of interventions to regulate breathing through capnographic feedback methods (24). Although the questionnaire has not been validated for use in adults with asthma (27) or in children, it does appear to be able to detect a cluster of symptoms that can be characterized as relating to the breathing-related effects of stress and anxiety.

**Table 1:** Prevalence studies of dysfunctional breathing identified by Nijmegen scoring in asthmatics.

**Study setting**	**Age**	**Study size**	**Age differences; Mean (SD)**	**Prevalence and sex differences**	**Comments**
UK General Practice (*N* = 7033) (17, 18)	17–65 years	71% of 307 asthmatics provided completed questionnaires	Dysfunctional breathers (DB) were younger: 44.8 years (14.7) vs. 49.0 years (13.8) *P* = 0.05	Overall prevalence; 29%. 35% of females and 20% of males Female:male, 73:27 (*P* = 0.016)	Patients were affected equally across all levels of severity. A follow up study found DB in 9.5% of non-asthmatics (14% of females and 2% males surveyed).
Greece Secondary care mild to moderate asthmatics (19)	20–68 years	162 participants; 94 female, 68 male	Not reported	Overall prevalence; 34% 46.8% of females and 14.7% males Female:male, 81:19	DB was more common in asthmatics that were moderate (72.9%) vs. mild (27.1%) and uncontrolled (81.4%) vs. controlled (18.6%).
Scotland secondary care “problem asthma clinic,” 76% of patients on BTS step 4 or 5 (20)	13.5–83 years	102 participants; 72 female, 30 male	Not reported	Overall prevalence 64%	Nijmegen scores were related to poor asthma control but more significantly related to quality of life measures.
Romania secondary care asthma clinics (21)	Mean age 35 years	91 participants; 47 female, 44 male	No significant age difference: 37.9 years in DB vs. 34.4 years	Nijmegen score prevalence: 29.7%, progressive exercise test prevalence: 17.6% 38% of females and 7.3% of males Female:male, 82:18	Severe asthma, lack of control and anxiety all associated with DB
Sweden secondary care clinic (22)	Mean age 47 years	25 participants; 19 female, 6 male	Not reported	Nijmegen score positive: 20%	Patients had well-controlled asthma
Spain secondary care clinic (23)	Mean age 47 years, 15–69 years	157 participants; 96 female, 61 male	DB were older: 49 years (24) vs. 42 years (17) *P* = 0.014	Overall prevalence 36% 47% of females and 19.6% of males Female: male, 79:21	Patients with DB experienced all asthma symptoms more acutely. They had more anxiety but no difference in overall asthma severity
Italy school setting (25)	11–14 years	120 asthmatics identified amongst 760 children (15.8%) 47 female, 73 male	No differences	Overall prevalence 25.8% 36.2% of females and 19.2% of males Female:male, 55:45	Asthma was more common amongst males. Amongst non-asthmatics, DB was found in 2.5% (4.1% of females and 0.9% of males surveyed).
Netherlands secondary care asthma clinic (26)	Mean age 10.4 years	206 consecutive asthmatics seen; 96 female, 144 male	No differences	Overall prevalence 5.3% 12% of females and 2.1% of males Female:male, 73:27	Asthma was more common amongst males. There was a strong dose dependent association between DB and asthma control

**Table 2:** The Nijmegen Questionnaire, Please circle the number in the column that best represents what you have felt recently^*^.

	**Never**	**Rarely**	**Sometimes**	**Often**	**Very often**
Chest pain	0	1	2	3	4
Feeling tense	0	1	2	3	4
Blurred vision	0	1	2	3	4
Dizzy spells	0	1	2	3	4
Feeling confused	0	1	2	3	4
Faster or deeper breathing	0	1	2	3	4
Short of breath	0	1	2	3	4
Tight feelings in the chest	0	1	2	3	4
Bloated feeling in the stomach	0	1	2	3	4
Tingling fingers	0	1	2	3	4
Unable to breathe deeply	0	1	2	3	4
Stiff fingers or arms	0	1	2	3	4
Tight feelings round mouth	0	1	2	3	4
Cold hands or feet	0	1	2	3	4
Palpitations	0	1	2	3	4
Feelings of anxiety	0	1	2	3	4
Subtotals					
Total					

* *A total score of 23 or more has been used to screen for dysfunctional breathing in a UK community setting (17). Cut-off scores to detect “abnormality” will depend on a comparison with normal values in the same setting and culture in which the questionnaire is used*.

Hyperventilation, as identified with this tool, is recognized as being common and occurs across the whole spectrum of asthma severity. It is more common in those with more severe disease and those with poor control (19, 26). Those studies that have investigated psychological co-morbidities, report that stress and the increased perception of more typical asthma symptoms occur more commonly in hyperventilators (21, 23). Such patients were also more likely to have acute asthma attacks.

All studies report that hyperventilation is more common amongst females vs. males and this sex difference tends to increase through childhood into early adult life. The only study in which asthma was more common amongst boys included a younger age group but, as in all other studies, the percentage of girls with asthma who also had hyperventilation was much higher than boys with asthma and hyperventilation (25).

It is not clear whether hyperventilation is more common in adults. A school study from Italy (25) produced comparable data to that in adult primary care, but a secondary care study from the Netherlands found far less dysfunctional breathing compared with adult data (26). Societal differences in reporting might be an important factor limiting the extent to which the Nijmegen questionnaire identifies abnormalities globally and might also limit the generalisability of such findings (28).

EILO is more common in adolescents and younger adults. It has been described in 26.9% of pediatric referrals to secondary care respiratory services, who were thought to have exercise induced asthma (29). Two population based studies suggest that it can occur in between 5.7 and 7.5% of none asthmatic adolescents (30, 31). Most studies also suggest a female predominance (15).

Adult studies have characterized a very severe phenotype of ILO in which symptoms typically occur without provocation. Amongst a hospital based series of 95 cases, 56% were diagnosed as also having asthma and 28% had suffered episodes needing endotracheal intubation to control symptoms (32). Two studies of adult asthmatics attending secondary care services report prevalences of 19 and 50% (33, 34). The study in which half of the asthmatics had this problem were identified using a novel computerized tomography imaging technique to none invasively assess laryngeal movements.

Poor discrimination between exercise induced inspiratory symptoms and exercise induced bronchoconstriction in adolescents and young adults, might be contributing to the over diagnosis of asthma in these age groups.

## Diagnostic Considerations (See Table 3)

Symptoms relating to dysfunctional breathing need to be differentiated from symptoms due to other causes. These include undiagnosed respiratory, cardiac or metabolic diseases associated with breathlessness, a lack of physical fitness, panic disorders whereby symptoms are more obviously a part of direct manifestations of anxiety, simply reaching physiological limits when exercising and the less common occurrence of wilfully fabricated or induced illness either by the patient or by proxy during childhood.

**Table 3:** Dysfunctional breathing in asthma: adults vs. children.

	**Children/young people**	**Adults**
Breathing pattern disorders	Girls more often affected and more common in those with psychological co-morbidity	Present across all asthma types and age groups. Women more often affected and those with more severe asthma and/or poor control and/or psychological co-morbidity.
Exercise inducible laryngeal obstruction	Exercise induced breathlessness poorly responsive to asthma treatment. Adolescents of either sex but more commonly girls who are elite athletes or “A” grade students but usually little in the way of psychological co-morbidity	Young adults predominantly as described in adolescents.
Inducible laryngeal obstruction	Limited evidence, but cases similar to that seen in adults have been described and occurring in increasingly younger age groups.	More common in women. Unprovoked asthma, treatment resistant, acute attacks resulting in escalation to high levels of treatment. High levels of physical and psychological morbidity.

Increasing breathing difficulties with prolonged inspiration, throat tightness, stridor and wheeze in the cervical region is highly suggestive of ILO in all age groups. A lack of response to more conventional asthma treatment is also indicative. Clinical clues suggesting the possibility of breathing pattern disorders include chest pain or discomfort with no other obvious cause, very short expiratory breath-holding times (e.g., < 20 s), feelings of not being able to take a deep breath, the abrupt onset of breathlessness with no obvious cause and the recognition that getting anxious is a trigger of respiratory symptoms (7).

Lactic acidosis after high doses of beta-2-agonists can of itself cause hyperventilation and further complicate a picture of acute asthma made worse by associated tremor and tachycardia which might compound feelings of anxiety (35).

Unfortunately there is no agreed diagnostic work up for this group of conditions. Screening tools for ILO have been suggested but these have largely been developed to distinguish ILO from asthma rather than recognize the two conditions as co-morbidities (36, 37). The medical history can be usefully informative about EILO. Although psycho-social stressors are less of a feature amongst young people with this condition compared with those who have unprovoked symptoms, they are typically “A” grade individuals, high performing athletes and commonly striving to fulfill parental or peer group expectations. Symptoms crucially peak during exercise or just after stopping, whereas exercise induced bronchospasm typically comes on 3–15 min after exercise. Whilst abnormal spirometry might be indicative of upper airway problems, it is poorly sensitive and should not be used in isolation for diagnosis (38). Typically there is inspiratory flow limitation, but there might also be a plateau in the expiratory flow rate (39). Laryngoscopy, performed during increasing levels of exercise to provoke symptoms, is regarded as the gold standard test, but diagnostic facilities are not widely available and can be difficult to perform in younger age groups (40).

Getting patients to perform voluntary over breathing challenge tests were recommended as a means of reproducing symptoms to support a diagnosis of hyperventilation (41). Initially, this was thought to occur through induced hypocapnia. However, subsequent studies have suggested that, in the majority of patients, alterations in feelings of anxiety and their central effects on neuro-muscular control of breathing are more important determinants of symptoms than respiratory alkalosis and such tests are no longer in common use (42, 43).

Hyperventilation is commonly screened for in asthma clinics using the Nijmegen questionnaire. The questionnaire matches up fairly well with more sophisticated diagnostic testing, such as graded exercise challenge tests (21). The creators of the questionnaire suggest that when used in clinical practice, it should be in conjunction with more objective measures of assessment (44). However, breathing assessments can be difficult as the use of mouthpieces and breathing circuits can directly alter breathing patterns. New technologies, such as structured light plethysmography, might help to better define breathing patterns in the future (45).

In an out-patient setting, there is usually little to find on clinical examination. If symptoms are present, it might be possible to differentiate upper from lower airway obstruction, but this can be difficult. Observing an abnormal breathing pattern might also be usefully informative, but young children in particular commonly breathe in strange ways when their chest is auscultated. Getting the patient or their family to use a mobile phone to capture episodes of abnormal breathing can sometimes be useful (4, 46).

## Treatment

An essential pre-requisite to treating dysfunctional breathing is to ensure optimal control of underlying asthma. This can be challenging given how similar respiratory symptoms occur in both problems and the need to contain the over use of medication.

Once problems, such as EILO and hyperventilation are identified, a clear explanation and reassurance about the nature of the problem can sometimes be effective in ameliorating symptoms. In the case of EILO, recordings of the larynx at endoscopy or direct visual feedback at the time of the procedure can be highly effective in explaining the cause of symptoms and the use of measures to overcome them (15). The optimal approach to ongoing treatment of (E)ILO is unclear (47). Many interventions have been suggested, but have only been studied in small, uncontrolled trials (48). The prognosis is also far from clear with conflicting case reports although those who have symptoms with no identifiable physical triggers (ILO) appear to do poorly (49). Most reviewers recommend the input of speech therapy services that have developed an interest and expertise in treating this problem and the use of inspiratory muscle training exercises. One retrospective study including adults and children suggested the benefits of inhaled anticholinergic agents in preventing exercise related problems (50). Laser supraglottoplasty has been used in highly selected cases with favorable results (40).

The intervention most commonly used for breathing pattern disorders is breathing retraining exercises. Pediatric studies are limited to reports of case series (51), but a large adult clinical trial in which asthmatics were taught by a trained physiotherapist or used a self-help online programme, reported significant improvements in quality of life scores compared to placebo (52). A smaller randomized controlled trial also reported positive results (53). A number of adult studies evaluating yoga and including yoga breathing techniques have shown small improvements in quality of life in unselected populations of asthmatics (54).

A pediatric service has reported a case series using individualized field testing protocols to characterize exercise related breathing problem in asthmatics and included the use of laryngoscopy to identify EILO as well as breathing pattern disorders thus facilitating individually tailored care plans (55). A pediatric respiratory physiotherapist led clinic designed to specifically address dysfunctional breathing problems has reported significant improvements in quality of life outcomes in support of this approach to treatment (56).

Whilst there is a good scientific rationale for psychological interventions to treat dysfunctional breathing, it is difficult to carry out well-designed studies in this area and there is little supporting evidence for this approach in any age group (57, 58). Suggestion therapy has been shown to be highly effective in young children with habit cough using a bed sheet as a bandage to strap and heal the chest, but a similar device has not been used for dysfunctional breathing in asthma (59).

Evaluating how psychotherapy might impact on dysfunctional breathing is compounded by the many ways in which environmental stressors might result in airway symptoms and associated confounders, such as poor adherence and poor lifestyle choices. These problems typically increase during adolescence and continue into early adult life. Stress increases the individual sensitivity to changes in airway caliber (60). It has also been shown to induce clinically significant bronchoconstriction in up to 40% of asthmatics under experimental conditions and asthmatics have been shown to develop increased indices of airway inflammation as a direct result of stress inducing challenges (61, 62). Research into psychological interventions in adults with asthma is inconclusive (63), but there are suggestions that interventions, such as cognitive behavioral therapy and mindfulness based stress reduction might improve both anxiety scores and asthma control. There is even less evidence for this type of psychological intervention in children and adolescents and high quality clinical studies with clearly defined outcomes are needed in this area (64).

## Conclusions

The diagnosis and treatment of dysfunctional breathing has mostly evolved through observational experience and a growing realization about the importance of this problem in all age groups. Further studies might usefully identify the extent to which the increasing emergence of this clinical problem is impacting on asthma morbidity and in particular during adolescence and early adult life. Recent epidemiological studies have characterized how asthma becomes more prevalent and severe after puberty and particularly in women (65). These changes parallel the emergence of dysfunctional breathing as an increasing problem in asthmatics.

Further studies are needed to help define the optimal approach to treatment in all age groups and to clearly delineate the long-term outcomes for different types of dysfunctional breathing across the lifecourse. Controlled trials have shown that many adults with asthma can benefit from breathing retraining programmes, most probably as a result of correcting breathing pattern disorders. Similar trials are urgently needed to assess the effectiveness of such interventions in children and adolescents.

## Author Contributions

All authors listed have made a substantial, direct and intellectual contribution to the work, and approved it for publication.

### Conflict of Interest Statement

The authors declare that the research was conducted in the absence of any commercial or financial relationships that could be construed as a potential conflict of interest.
